# Polycarbonate Masters for Soft Lithography

**DOI:** 10.3390/mi12111392

**Published:** 2021-11-13

**Authors:** Filippo Amadeo, Prithviraj Mukherjee, Hua Gao, Jian Zhou, Ian Papautsky

**Affiliations:** Department of Biomedical Engineering, University of Illinois at Chicago, Chicago, IL 60607, USA; famade3@uic.edu (F.A.); pmukhe5@uic.edu (P.M.); hgao22@uic.edu (H.G.); jzhou88@uic.edu (J.Z.)

**Keywords:** soft lithography, replica molding, polycarbonate, polymer masters, microfluidics

## Abstract

Fabrication of microfluidic devices by soft lithography is by far the most popular approach due to its simplicity and low cost. The approach relies on casting of elastomers, such as polydimethylsiloxane (PDMS), on masters fabricated from photoresists on silicon substrates. These masters, however, can be expensive, complicated to fabricate, and fragile. Here we describe an optimized replica molding approach to preserve the original masters by heat molding of polycarbonate (PC) sheets on PDMS molds. The process is faster and simpler than previously reported methods and does not result in a loss of resolution or aspect ratio for the features. The generated PC masters were used to successfully replicate a wide range of microfluidic devices, including rectangular channels with aspect ratios from 0.025 to 7.3, large area spiral channels, and micropost arrays with 5 µm spacing. Moreover, fabrication of rounded features, such as semi-spherical microwells, was possible and easy. Quantitative analysis of the replicated features showed variability of <2%. The approach is low cost, does not require cleanroom setting or hazardous chemicals, and is rapid and simple. The fabricated masters are rigid and survive numerous replication cycles. Moreover, damaged or missing masters can be easily replaced by reproduction from previously cast PDMS replicas. All of these advantages make the PC masters highly desirable for long-term preservation of soft lithography masters for microfluidic devices.

## 1. Introduction

Soft lithography is the most common approach for rapid and low-cost fabrication of microfluidic devices for medicine, biology, and chemistry. Soft lithography relies on casting of elastomers, such as polydimethylsiloxane (PDMS), on master molds fabricated from photoresists on silicon substrates [1,2]. These silicon-photoresist masters (SPMs) offer excellent feature resolution and are conventionally fabricated by photolithography in a cleanroom using SU-8 negative photoresists. SPM fabrication generally requires significant user expertise, as many of the steps are manual, as well as high material and equipment costs. Although alternative approaches using dry-film photoresists have been reported [3,4], they are not yet widely accepted. However, photoresists generally perform poorly as structural materials due to delamination at the photoresist-silicon interface after a number of heating-cooling cycles due to repeated PDMS casting. This issue is more prominent for thicker resists and higher aspect ratio structures [5]. Furthermore, the silicon wafer itself is brittle and can shatter if too much force is accidently applied when cutting out PDMS replicas. Consequently, SPMs have a limited casting lifetime. Mechanical milling and 3D printing have emerged as attractive alternatives to master fabrication. However, high roughness of the generated surfaces and the limitations in feature resolution, coupled with high cost of required equipment, limit their use.

A promising approach that overcomes issues associated with fabricated masters is to copy the existing master. In this approach, an elastomeric master formed by copying the SPM is then used to fabricate a rigid copy mold through an additional replication process. These approaches include epoxy [6,7], polyurethane [8], polystyrene [9,10], and polyvinylsiloxane [11]. The cured polymeric master can then be used to cast PDMS microfluidic devices by soft lithography. However, such masters are expensive, require curing equipment, and large-area fabrication may be challenging due to the need for a uniform UV illumination [8]. Further, during the pouring and the degassing steps, the PDMS micro features, especially high aspect ratio ones, can get distorted by the instantaneous drag force exerted on them by the uncured polymer [12]. Moreover, some UV curable resins have low heat deflection temperature that imposes a constraint on the PDMS curing temperature, increasing the curing time and reducing the fabrication throughput [12]. Ultimately, polyurethane solutions cannot be degassed after being poured on the PDMS mold [8].

In this work, we overcome these limitations by replicating soft lithography masters in polycarbonate (PC) thermoplastic. The process, first reported by Sonmez et al. [12], involves softening of PC sheets by raising temperature above glass transition (Tg) and allowing them to reflow on PDMS mold. Once cooled and separated, the resulting PC masters (PCMs) faithfully replicate the PDMS structures. In essence, the approach is the reverse of the hot embossing process with PDMS tools that we [13] and others [14] have reported previously, but without force application to avoid distortion of the microfeatures. Here we demonstrate a much simpler process that does not require UV curing or plastic molding, and can be accomplished with a single vacuum oven in just a few hours (<6 h). There are no practical limitations to the mold size or thickness that can be replicated. We applied the PCM technique to replicate various types of microfluidic device SPMs. Following quantitative characterization, we show that both low and high aspect ratio microchannels spanning most of the length of a 3-inch microscope glass slide can be accurately replicated.

## 2. Experimental Methods

### 2.1. SPM Fabrication and Soft Lithography

Soft lithography masters were fabricated by laminating dry film photo-resist on a 3-inch silicon wafer and exposing it to UV light through patterned masks, as described by Mukherjee et al. [3]. Several patterns were transferred to the masters: straight and spiral rectangular microchannels having different aspect ratios, micro post arrays, and micro well arrays. The elastomeric replicas of silicon-photoresists (SPM) masters were realized by standard soft lithography using PDMS (Sylgard 184, Dow Corning): monomer was mixed in a 10:1 mass ratio with its curing agent, degassed, poured on the SPM, degassed again, and cured at 75 °C for 2 h. PDMS replicas were peeled from the masters, cleaned with tape, and used as molds.

### 2.2. PCM Fabrication

**Figure 1:**
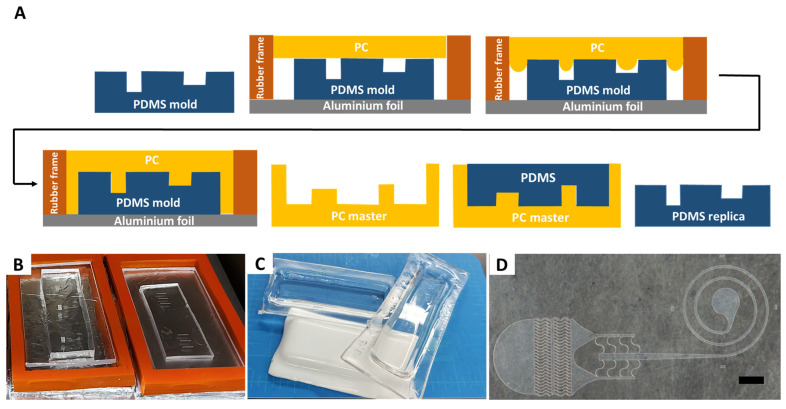
Polycarbonate master fabrication process. (**A**) Schematic diagram of the molding process. A PDMS soft lithography replica of the original master is used as a mold. Dried PDMS molds are placed in a vacuum oven over an aluminum foil with a heat resistant rubber frame around and a dried PC slab on top. Following baking process at 220 °C for 2–4 h, PC softens and re-flows around the microscale features. Once cooled to room temperature, PDMS mold is removed, and a rigid and durable PC master is obtained. The PC master is then used in multiple replication steps in place of the original master to cast PDMS replicas. (**B**) Photograph of the PC sheet on top of PDMS mold, inside a high temperature resistant rubber containment frame. (**C**) Examples of fabricated PC masters. (**D**) Bright field image of a representative PC master, illustrating an input port with filter and a spiral channel of a microfluidic device; scale bar is 1 mm.

Figure 1A illustrates the key steps of the PC heat molding process. A 3/32 inch (~2.5 mm) thick transparent, white and black polycarbonate sheets were purchased from McMaster-Carr (Elmhurst, IL, USA) and cut into slabs wider than the PDMS molds using a Dremel^®^ rotary tool equipped with a cut-off wheel. After cutting, the edges of the slabs were sanded using sandpaper. The protective film was removed from both sides of the PC slabs that were then sprayed with IPA and blow dried to remove any PC dust originated during the cutting process. The PDMS molds were cleaned with adhesive tape. Both the cleaned PC slabs and PDMS molds were placed to dry in a vacuum oven (ThermoFisher Scientific, Waltham, MA, USA) at 125 °C for 2 h; the vacuum pump was kept on during the process and lowered the pressure to ~49 mm Hg. As an alternative, the drying process can be performed overnight on a hotplate at 125 °C. Once dry, the PDMS molds were arranged along with a high temperature resistant 1/4 inch thick rectangular silicon rubber gasket, and with the PC slabs placed on top, in rows on the oven shelf covered with aluminum foil, and in its center, where the temperature is more uniformly distributed. The oven temperature was then set to 220 °C and PC was baked for 2–4 h depending on the features density and aspect ratio. After baking, the molds were removed from the oven and allowed to cool at room temperature for 30 min. PC was then delicately freed from the PDMS mold using metal tweezers, cleaned with IPA, and used to fabricate PDMS microfluidic devices by soft lithography.

### 2.3. Process Variations

To replicate triangular cross sections channels, a micromilled brass plate was used as a master for PDMS soft lithography. In this case, since the triangular features were engraved in the brass, to obtain a PDMS mold with empty triangular features that could be filled by PC during baking, a PDMS double casting step was necessary. Hence, the PDMS replica obtained by soft lithography from the micromilled brass plate was siliconized by coating its surface with SigmaCoat (SL2, Sigma-Aldrich, St. Louis, MO, USA) under a laminar flow hood. After 1 h exposure, it was washed with deionized water and once dry it was encased in aluminum foil and used as a mold for PDMS soft lithography. After curing, the top PDMS part was delicately peeled off from the bottom part and used as a mold in the heat molding process.

To obtain a PC master with rounded structures by molding it on rectangular features, the baking temperature was set to 190 °C and the baking time reduced to 20–60 min, depending on the desired rounded features depth, in order to slow the process and melt PC more uniformly across the mold area. The baking set up was also modified as follows: a microscope glass slide and another aluminum covered brass plate were placed on top of the PC slab. The 8 cm × 5 cm brass plate had a mass of 108.2 g, an area of 40 cm^2^, and allowed to obtain uniform round features across the mold by applying a gentle pressure.

### 2.4. Microscope Imaging and Data Analysis

An optical microscope (Olympus IX73, Olympus America Inc., Lombard, IL, USA) connected to a high-resolution sCMOS camera (Zyla 5.5, Andor, Concord, MA, USA) was used to acquire top and cross-sectional images of the PDMS replicas. To acquire top view images, the PDMS replicas were cleaned with tape and placed on a microscope glass with the features facing down. A 4× objective was used to obtain bright field images. Cross-sectional images were taken by slicing the PDMS replicas vertically with a razor blade, obtaining a few mm thick slices that were laid down on the microscope glass. In this case, 40× objective was used to obtain bright field images. For straight microchannels, sections were cut orthogonal to the channel every 5 mm along the length. For spiral channels, sections were cut at every 90° turn. For post and well arrays, the sectioning was carried out diagonally to the array direction to increase the number of sectioned features. The dimensional measurements of the PDMS microfeatures were performed manually using CellSense imaging software (Olympus America Inc., Lombard, IL, USA). The measured data were stored, analyzed, and plotted in Origin Pro 2021 (OriginLab Co., Northampton, MA, USA). Both the PDMS replicas of the original master and of the PC master were imaged to measure and compare the dimensions of their microfeatures to quantitatively validate the replication accuracy of the process. A quantitative analysis was performed by analyzing the measurements of the characteristic dimensions of the microfeatures: the height and the width of rectangular channels, the spacing and diameter of post arrays, the depth of microwells, the depth, and the base of triangular channels. For each class of microfeatures, the imaging and measuring of features was repeated five times from different replicas, cast from the SPM master and from different PC masters. To quantitatively assess the impact of the key process parameters on the formation of bubbles in PC, images of PC masters were acquired using a digital microscope with a zoom lens (ScienceScope International, Chino, CA, USA). The images were processed on ImageJ software to obtain a binary image from which the percentage of area of the master occupied by bubbles was computed using the particle analyzer tool of the software.

## 3. Results and Discussion

### 3.1. PCM Fabrication

The PCM fabrication process starts with making a PDMS replica of the original master. The process is schematically illustrated in Figure 1A. The approach yields a monolithic master, directly addressing the delamination and poor structural integrity of SPMs. Herein, we used SPMs fabricated by standard photolithographic techniques or by dry photoresist, as we reported earlier. However, instead of using SPM to generate device replicas in PDMS, as in a standard soft lithography process, we used the SPM to cast PDMS once, and then used it to fabricate a PCM that clones the original SPM. Alternatively, 3D printed or micromilled masters can be used to begin the process. Ultimately, multiple PCMs can be generated, allowing for cost-effective scale up of masters and devices.

A rectangular rubber gasket was placed around the PDMS replica to form perimeter of the mold (Figure 1B). Gaskets of custom size and thickness can be cut from a sheet of rubber and reused in multiple processes. A piece of a cut PC sheet was then placed on top of the PDMS replica and heated in the vacuum oven. The baking time depended on the aspect ratio and on the minimum spacing of the mold micro features and ranged from a minimum of 2 h for low aspect ratios and spaced features, to 4 h for high aspect ratio and dense features. It is preferable to conduct the baking process in a well-ventilated room since PC fumes can build inside the oven and release when opening the oven door.

PCM fabrication can be used to generate masters of varying thickness and area. We have created plastic masters of a single device the size of a standard microscope glass slide (Figure 1C) as well as masters encompassing a larger 3 in wafer area. For microfluidic applications, the form factor of a microscope glass slide (1 in × 3 in) is the most useful due to the sealing process of PDMS replicas subsequently formed using these masters. Both large area and small area topology can be replicated in a single master (Figure 1D), giving the approach added flexibility. Furthermore, the fabricated PCMs have Rockwell R hardness of 118 and tensile strength >8900 psi, making them resistant to abrasion and fracture and sufficiently robust for daily handling in a laboratory [15]. In addition, PCMs exhibit good chemical resistance against diluted acids, aliphatic hydrocarbons, and alcohols, but are attacked by diluted alkalis and aromatic and halogenated hydrocarbons [16]. PCMs are also thermally stable up to 135 °C, making them compatible with the wide range of curing temperatures of the PDMS soft lithography process.

PCMs can be fabricated in a variety of colors, from natural (transparent) to black, stemming from polycarbonate sheets being available in a wide range of shades. In its natural form, PC is an extremely clear plastic that can transmit over 90% of light [17], yielding PCMs that are as clear as glass and are convenient for observation of the PDMS patters during casting. The use of color (e.g., white PCM in Figure 1C) can be advantageous for color-coding and simplicity of attributing masters to a particular project or to lab personnel. However, it must be noted that pigment additives can impact the mechanical and rheological properties of PC [18,19]. Indeed, recent work suggests that addition of ~5% of pigment (by volume) can modify the melt flow index (MFI)—a measure of the ease of flow of a thermoplastic polymer—by as much as 16% [19]. Such changes in rheology would necessitate process optimization for each color. Most of the work herein was carried out using the natural PC sheets, as the transparent nature of the material also permitted better observation of the process.

**Figure 2:**
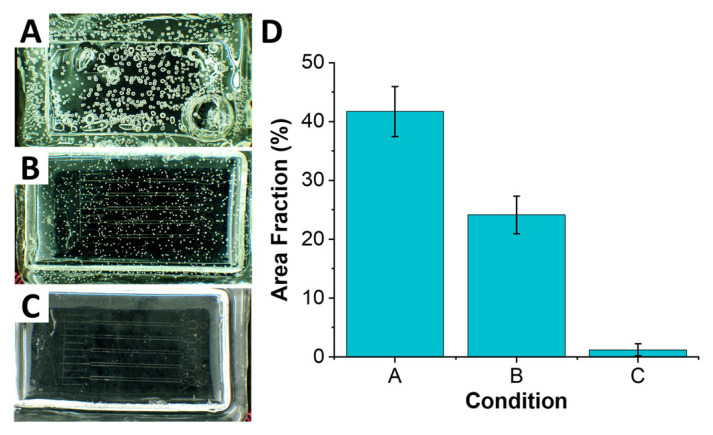
Impact of the drying process and thermal stress on fabrication of PC masters. (**A**) PCM not dried and subjected to thermal stress. (**B**) PCM not dried and not subjected to thermal stress. (**C**) PCM dried for 2 h at 125°C and vacuum at ~49 mm Hg and not subjected to thermal stress. (**D**) Quantitative comparison of the area occupied by bubbles in PCM in scenarios (**A**–**C**) (*n* = 3). Drying PC before baking and avoiding thermal stress by placing PC in the oven below its glass transition temperature were found to be essential to avoid the formation of bubbles.

The drying phase of the fabrication protocol proved to be one of the most critical steps since both PC [20] and PDMS [21] are hygroscopic and can absorb moisture from air. It is this absorbed moisture that is thought to be the cause of formation of air bubbles throughout the sheet when heated above 125 °C (Figure 2A). These air bubbles can affect the correct replication of microfeatures, as it is well-known in hot embossing and soft lithography [14,22]. Thus, both PC sheets and PDMS molds were dried below Tg prior to the molding process. The drying step was performed for 2 h in a vacuum oven at 125 °C; a vacuum at ~49 mm Hg was used to aid the process. Sonmez et al. [12] reported drying PDMS molds at 60 °C for 24 h; this substantially longer drying time was needed due to using base to curing agent ratio of 5:1 to fabricate PDMS molds. The higher fraction of curing agent yields a stiffer PDMS material, which is desirable for a molding process especially of higher aspect ratio structure. However, this also makes PDMS less gas permeable, making the drying process extremely long and not always effective [23]. We found that higher gas permeability of the conventional 10:1 PDMS mixture allowed for a much more rapid drying process, while retaining ability to replicate high aspect ratio features (as we discuss in the next section).

In addition to the drying phase, we found that thermal stress plays a role in formation of air bubbles throughout the polymer. The thermal stress arises due to rapid change in temperature. In our case, placing PC sheet into oven preheated to 220 °C baking temperature yielded a significant and rapid change from room temperature, which resulted in formation of numerous air bubbles. One of the reasons for this, as discussed above, is the rapid vaporization of moisture trapped within the polymer, stemming from its hygroscopic nature. As water vapor expands and escapes from polymer above its glass temperature, it forms small bubble cavities in the compliant polymer. We found that starting the process below Tg, at 125 °C, and ramping it up to the 220 °C baking temperature (at ~5 °C/min) allowed vapor to escape with significantly fewer (nearly 2× fold) distortions to the polymer (Figure 2B). Coupling the temperature ramping with the drying step yielded the best results (Figure 2C) with nearly no bubbles forming.

The baking process was performed in the same vacuum oven as in the drying phase, without removing components. The baking temperature was set at 220 °C, selected to exceed the Tg of PC (~150 °C) but remain below the thermal degradation temperature of PDMS (~280 °C). The baking time was found to depend on the density and aspect ratio of the microfeatures, as it was desirable for the PC melt to fill the PDMS mold features. For the widely spaced and low aspect ratio features (AR < 1.5), a 2 h bake time yielded accurate replication. For higher aspect ratio or dense structures, a longer 4 h baking time was necessary.

After baking, the molded PCM was allowed to cool to room temperature for 30 min (~6.5 °C/min) and was separated from PDMS mold. Sonmez et al. [12] coated PDMS molds prior to the drying and baking steps with tridecafluoro-1,1,2,2-tetrahydrooctyl-1-trichlorosilane (TFOCS) to lower surface energy and improve mold release. However, we found that a mold release coating was not necessary as PDMS molds and PCMs detached effortlessly. It is possible that higher pattern density or higher aspect ratio microfeatures than those testing in this work may necessitate use of a release agent. In such cases, Sigmacoat silanization agent can be used. Sigmacoat is a solution of a chlorinated organopolysiloxane in heptane that reacts with surface silanol (Si–OH) groups to produce a hydrophobic film and is commonly used to aid mold release is soft lithography. The coating process is rapid, only requiring a few minutes inside a fume hood, and is thus much faster than the 2 h coating process reported by Sonmez et al. [12].

The formed PCMs can now be used to cast PDMS using the standard soft lithography process. We have used PCMs to generate PDMS replicas at least 20 times without any visible degradation. To ensure a dust-free surface, PCMs can be cleaned with IPA and dried using an air gun before each PDMS casting, due to the excellent chemical resistance of PC to IPA. When demolding the cured PDMS replicas from the PCM, it is best to avoid touching the patterned areas to minimize chance of scratches. Finally, we also found that PDMS molds themselves can be used multiple times to fabricate multiple PCM copies.

### 3.2. Cross-Sectional Characterization

**Figure 3:**
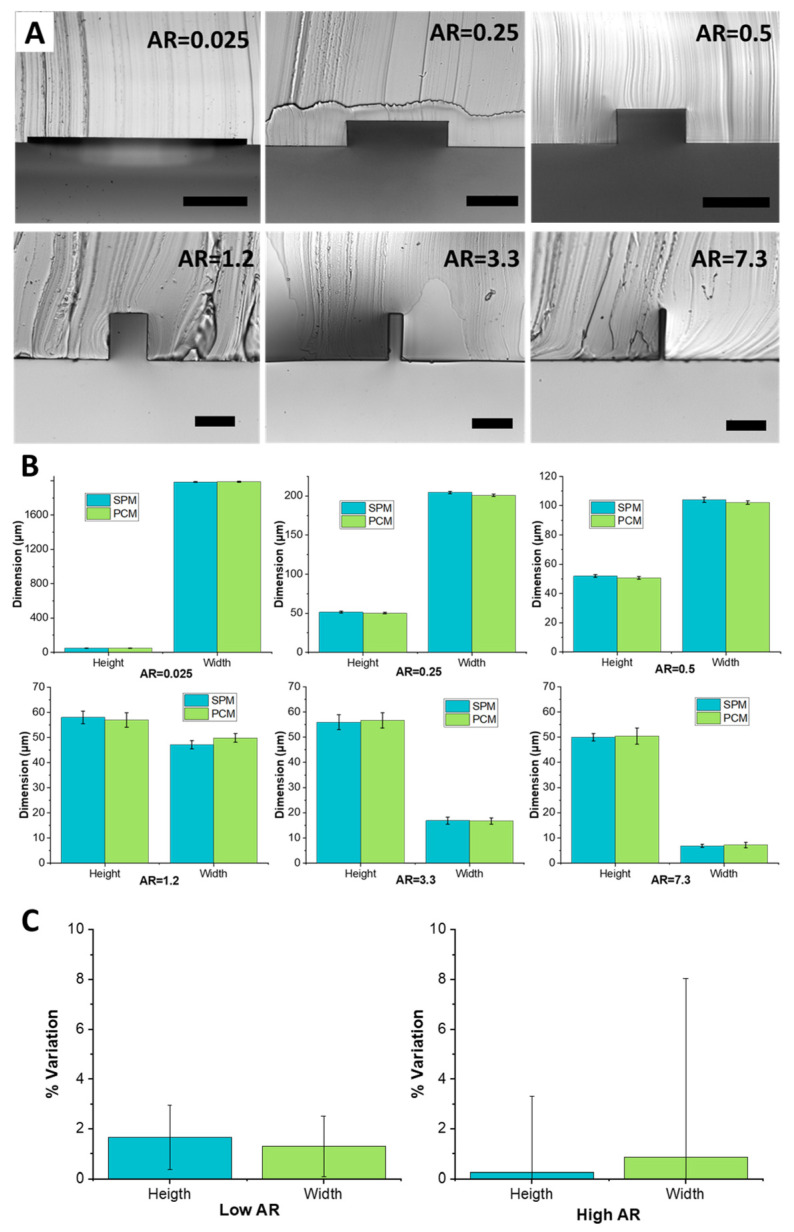
PDMS replicas of channels of various aspect ratios formed using PCMs. (**A**) Cross-sectional images of channels formed from PCMs. The channel dimensions were: 2000 µm × 50 µm (AR = 0.025, scale bar is 500 µm), 200 µm × 50 µm (AR = 0.25, scale bar is 100 µm), 100 µm × 50 µm (AR = 0.5, scale bar is 100 µm), 58 µm × 47 µm (AR = 1.2, scale bar is 50 µm), 56 µm × 17 µm (AR = 3.3, scale bar is 50 µm), and 50 µm × 7 µm (AR = 7.3, scale bar is 50 µm). (**B**) Quantitative comparison of the cross-sectional dimensions (*n* = 5). (**C**) Variability of cross-sectional dimensions along the 4 cm channel length for low aspect ratio (AR < 1) and high aspect ratio (AR > 1) channels (*n* = 5).

Masters with different aspect ratios were fabricated to assess performance range. Figure 3A shows cross-sections of different aspect ratio channels, illustrating precise rectangular shape and perpendicular walls. The channels ranged from a 2 mm wide low aspect ratio channel to a 7 µm high aspect ratio channel. Specifically, the included channels were as follows: 2000 µm × 50 µm (AR = 0.025), 200 µm × 50 µm (AR = 0.25), 100 µm × 50 µm (AR = 0.5), 47 µm × 58 µm (AR = 1.2), 17 µm × 56 µm (AR = 3.3), and 7 µm × 50 µm (AR = 7.3). The images were obtained by forming PDMS replica of the PCM and then slicing through it to image its cross-section. Across all aspect ratios, geometries were faithfully replicated. The slender structures, at both low aspect ratio (AR = 0.025) and high aspect ratio (AR = 7.3) were fabricated without any distortion or deformation during the heat molding process.

Measurements from PDMS replicas show that the completed channels have dimensions within ±2 μm of the dimensions of the original PDMS mold (Figure 3B). For low aspect ratios (0.025 to 0.5) variation of height and width stayed within 1–2% of the original SPM dimension. For AR = 1.2, a slight decrease in the height and a slight increase of width of a few microns was observed. This variation stays within the 2% of the original dimensions. For AR = 3.3 the channel height saw an increase of 1 µm while the width remained constant. For AR = 7.3, the channel height and width variations were limited to 1 µm. The high closeness of channel dimensions casted from the SPM master and from the PC master confirms the high fidelity of the replication abilities of the process and the absence of any detectable shrinkage of PC features during cool down. The standard deviation of the data was low, although increasing with aspect ratio, suggesting low variability and thus high quality of replication. In addition, channels replicated from PCMs were constantly slightly smaller (~1–2%) in width and height than the original SPMs. Additionally, the PDMS surface around the bottom of the channel was smooth, suggesting that quality of the subsequent bonding step necessary to seal microfluidic channels should be unaffected by the PCM process.

Downstream channel uniformity of the PCM is another important factor that can impact microfluidic device performance and is an indicator of replication quality. The uniformity was measured by slicing the 4 cm long PDMS channel replicas at 5 mm increments. Figure 3C reports variation in channel height and width in the downstream direction. For low aspect ratio channels (AR < 1), the downstream variability in channel dimensions was <2%. For high aspect ratio channels (AR > 1), dimensional variability was even less, <1% on average, although variations from channel to channel were more significant as indicated by the error bars. Ultimately, the quality of replication was high.

### 3.3. Replication of Various Channel Geometries

**Figure 4:**
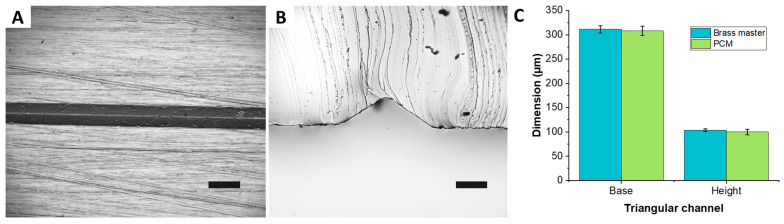
Replication of triangular channels. PDMS replicas of triangular micro channels formed from PC master; PC has been molded on a PDMS replica of a micromilled brass plate patterned with a 100 µm deep, 300 µm wide triangular channel (~113° apex). (**A**) Top view of the triangular channel; scale bar is 500 µm. (**B**) Cross section of the channel; scale bar is 100 µm. (**C**) Quantitative comparison of channels base and height with respect to the replica of the original master (*n* = 5).

To assess the ability of the PCM approach to reproduce non-rectangular channel geometries, an 8 cm × 5 cm micromilled brass plate was used to cast a PDMS mold of a microchannel with a triangular cross-section. The surface was patterned using a conic engraving tool to realize 4 parallel straight channels having different depths. Here, the smallest channel (depth of ~100 µm, base of ~300 µm, and apex angle of ~113°) was considered to assess the replication capabilities of the PCM heat molding process. Microscope images of PDMS cast from the PCM shows a well-defined and straight channel having cross section with sharp edges (Figure 4). Quantitative comparisons of the channel height and base show very low dimensional variations of four microns only (Figure 4C). Directly heat molding PC on the brass master turned out to be impractical due to inability of the metal mold to absorb air that fills the microfeature, yielding pockets of air trapped in between the PC and the bottom of the triangular microchannel. These air pockets were detrimental to the channels cross-sectional geometry. Other limitation of using micromilled metal masters included the high surface roughness that could affect the bonding quality to the glass substrate during subsequent microfluidic experiments, and high thermal conductivity that impacted the polymer thermal cycle. Ultimately, forming PCM from PDMS replicas yielded channels that replicated the triangular cross-section with high accuracy.

**Figure 5:**
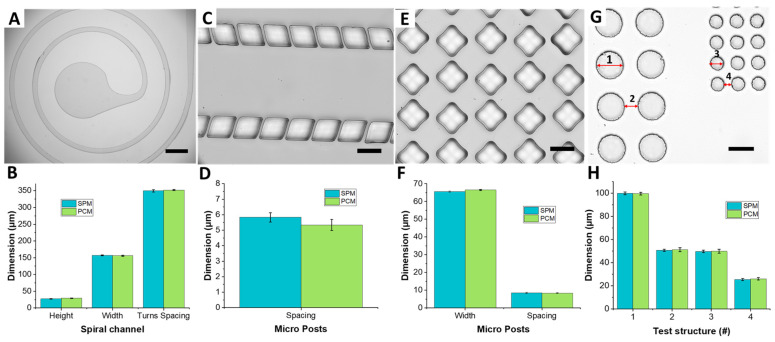
PDMS replicas formed from PC master of a microfluidic device having a spiral channel, micro post arrays having different spacing and shapes. (**A**) Top view image of the spiral channel covering an area of 30 mm^2^, channel cross section has a width of 150 µm and a height of 30 µm, and adjacent turns are spaced by 350 µm; scale bar is 500 µm. (**B**) Quantitative comparison of spiral channel height, width and spacing (*n* = 5). (**C**) Two parallel post arrays spaced by 5 µm; scale bar is 50 µm and (**D**) quantitative comparison of post spacing (*n* = 5). PC melt can flow in gaps as small as 5 µm during the heat molding process. (**E**) A matrix of staggered micro posts having minimum spacing of 8 µm; scale bar is 50 µm and (**F**) quantitative comparison of posts structures width and spacing (*n* = 5). (**G**) Circular pillar arrays of diameter 100 µm (Test Structure 1) and 50 µm (Test Structure 2), separated by 50 µm (Test Structure 3) and 25 µm (Test Structure 4) gaps; scale bar is 100 µm and (**H**) Quantitative comparison of Test Structures (*n* = 15).

To assess the PC replication capabilities of structures having large area, a microfluidic device with a low aspect ratio spiral channel was used. The spiral channel cross-section was 150 µm wide and 30 µm high (AR = 0.2). The spacing between the adjacent turns of the spiral was 350 µm, and the area covered by the spiral was 30 mm^2^. The top view image of the PDMS replica cast from PCM shows well-defined and highly concentric spiral channels (Figure 5A). The dimensional comparison of the PDMS replica of the PCM with the one cast from the original SPM shows that the spacing between adjacent turns variation stays within the 4% of the original dimensions (Figure 5B).

To demonstrate replication of dense post arrays that PC could correctly copy, a number of test structures were fabricated. These included a microfluidic device having two arrays of microposts at 5 µm spacing, a matrix of diamond microposts that are 65 µm wide and high and at 8 µm spacing, and arrays of circular posts (100 µm diameter at 50 µm spacing, and 50µm diameter at 25 µm spacing). By analyzing the microscope images of the PDMS micropost arrays, it was confirmed that PC can flow in between features spaced by as little as 5 µm, replicating micropost arrays of different geometries and spacing correctly (Figure 5C,E,G). Indeed, the averaged measurements show that the spacing between posts is replicated and varies within a reasonable experimental error of ~2–3 µm (Figure 5D,F,H). While the post spacing of 5 µm corresponds to the smallest feature replicated here, it is not necessarily the resolution of the process. In this work we optimized the process in terms of the required time and instrumentation. Previous work [12], however, demonstrated replication of ~1 µm features, which we believe is much closer to the resolution limit of our approach.

### 3.4. Fabrication of Rounded Features

Since during the baking process PC melt gradually flows into microfeatures to fill the mold shape, it may be possible to intentionally obtain rounded, partially-filled features by interrupting the process. One potential application of this is formation of microwells with rounded bottom for culture of cell spheroids or patient-derived organoids [24]. Having wells with rounded U-shaped bottom rather than the traditional flat bottom ones has the advantage of promoting cell-to-cell adhesion and formation of spherical cell aggregates (spheroids). Previously, we had resorted to the use of 3D printing to generate U-shaped wells [24], but were limited by printer resolution (~25 µm) and stepped cross-sectional profile. Outside cell culture, rounded features can be used as micro lenses in optical applications [25]. Consequently, we explored the possibility of making such features.

**Figure 6:**
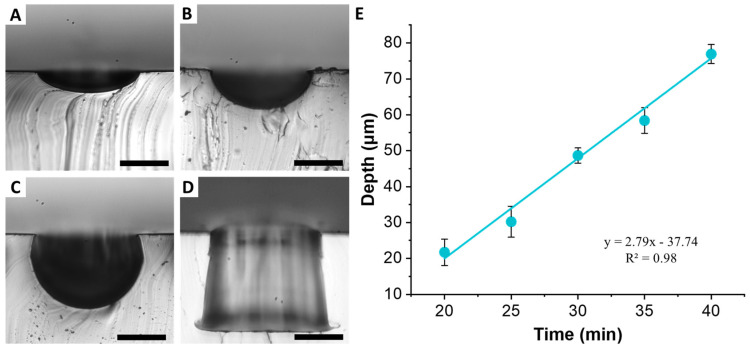
Formation of PCM with rounded features from PDMS mold. Cross-sections of formed rounded microwells after baking for (**A**) 20 min, (**B**) 30 min, and (**C**) 40 min; scale bar is 50 µm. (**D**) Full well replicated after baking for 60 min. (**E**) Relationship between baking time at 190 °C and microwell depth (*n* = 3) appears to be linear.

An array of cylindrical microwells 100 µm in diameter and 100 µm in depth was used to demonstrate formation of U-shaped structures. The baking time was the most important parameter to control, since it was necessary to stop the PC melt flow before the complete structure was replicated. Baking for 20 min allowed us to obtain shallow 21.7 ± 3.6 µm deep wells (Figure 6A). The 100 µm diameter well structure was preserved at the top. However, the bottom of the structure was clearly rounded, indicating that PC melt just started to flow into the well structure. Increasing bake time of 30 min yielded depth of 48.6 ± 2.1 µm (Figure 6B). Again, the top of the structure was well preserved, while the bottom exhibited a semicircular shape. Increasing time further to 40 min, resulted in depth of 76.9 ± 2.6 µm (Figure 6C). The complete replication of the cylindrical well occurred around 60 min (Figure 6D). Plotting the rounded well depth as a function of bake time reveals a linear relationship (Figure 6E). Regardless of the baking time, the diameter of the wells remained constant, although increased slightly from the original PDMS mold to 105.8 ± 2.8 µm, which is most likely due to the slanted side walls of the original well. These results suggest that PCMs of wells of different depth can be obtained from a single PDMS mold by simply by tuning the baking time and without the need to re-fabricate SPMs with multiple thicknesses.

In addition to modifying the baking process time, the baking temperature was lowered to 190 °C to increase PC melt viscosity to improve uniformity and slow the flow of PC inside the features. The uniformity can also be improved by adding a weight on top of the PC sheet during the molding process to flatten the PDMS-PC contact area. Indeed, since the process must be stopped before PC entirely fills the wells, any slight difference in the initial relative position of PC and PDMS across the mold area can bring to differences in the wells’ depths of up to tens of microns. For this, an 8 cm × 5 cm brass plate with a mass of ~108 g was placed on top of a PC sheet at the start of the replication process. The added weight improved uniformity of wells considerably.

## 4. Conclusions

The PCM approach presented herein allows to overcome the limited lifetime challenges associated with the conventional SPM fabrication methods and offers a number of additional advantages. This is in part due to the monolithic mold structure and in part due to the thermoplastic being a much harder material as compared to photoresist. Consequently, the PCMs can be re-used to cast PDMS devices nearly indefinitely. Indeed, we have generated more than 40 replicas from a single PCM over the course of six months. The inherent robustness of the PCMs not only increases master lifetime but also permits easy backside labeling through engraving, which does not wash or rub away. Additionally, PDMS molds and PCMs do not require salinization, as opposed to the majority of previously reported replica molding approaches, further simplifying fabrication process. The superior rigidity and thermal stability allow to obtain higher aspect ratios with minimum dimensional variation.

The PCM fabrication process also offers a number of advantages over other previously-reported replica molding approaches [8,12], as we summarize in Table 1. The PCM approach permits quick replication of master molds in PC sheets, yielding masters in as little as 6 h, and can be accomplished without the use of sophisticated or costly equipment. The approach is quite simple and does not have the complexity of casting two-component epoxies or urethanes, or the need for multiple ovens at different temperatures. The smallest feature we replicated was 5 µm, although we believe the process resolution to be higher (based on previous work).

Although 3D printing has emerged as an attractive alternative to master fabrication due to the ability to fabricate complex multi-level geometries that can be used to form master molds for soft lithography [27], this application is in its infancy. Although 3D printing is becoming more and more affordable, the low-cost systems lack the resolution and are not capable of printing features below 15–25 µm with high fidelity. Additionally, the presence of layer deposition artifacts, which increase surface roughness, and the edge rounding effects further limit quality and the widespread use of 3D printing for microfluidics applications [27]. Finally, the resin formulations used in many 3D printers are not biocompatible and often contain compounds that interfere with polymerization of PDMS [28]. While 3D printing continues to evolve and is likely to overcome many of these challenges in the future, at present the PCM approach offers rapid and low-cost fabrication of smooth replicas.

In addition, the possibility of fabricating rounded features in PC offers an exciting opportunity that is not easily replicated with the conventional approaches. By controlling baking time, it is possible fill microfeatures only partially with PC melt. The end result is the formation of molds that feature rounded structures, arising from the laminar velocity profile of the PC melt during the baking process. The flow of PC melt in cylindrical wells is a function of time and follows a linear trend, permitting easy control of feature depth. Modeling the PC melt flow more accurately may allow further optimization of the molding process.

Ultimately, the PCM process presented herein offers a reliable alternative to fabrication of soft lithography masters. The approach is low cost, does not require cleanroom setting or hazardous chemicals, and is rapid and simple. The fabricated masters are rigid and survive numerous replication cycles. Moreover, damaged or missing masters can be easily replaced by reproduction from the previously cast PDMS replicas. All of these advantages make the PCM process highly desirable for long-term preservation of soft lithography masters for microfluidic devices.

## Figures and Tables

**Table 1:** Comparison of fabrication methods for soft lithography masters.

Key Features	Polymer Masters [8]	Polycarbonate Heat Molding Masters [12]	PCMs (Present Work)
**Non-Cleanroom Processing**	Yes	Yes	Yes
**Setup costs**	Low	Low	Low
Toxicity of materials and reagents *	Low	Very Low	Very Low
**Level of Expertise**	Low	Low	Low
**Process time**	~3 h	~30 h	4–6 h
Lifetime **	High	Very High	Very High
**Surface treatment**	No	For high aspect ratios	No
**Baking temperature**	-	230 °C	220 °C
**Replication shape & size**	Any shape, any size	Any shape, any size	Any shape, any size
**Aspect ratio range**	-	<7	<7.3
**Smallest replicated feature**	~2 µm	~2.5 µm	~5 µm
**Variability**	4%	3–5%	2%

* Based on product literature [15,26]; ** Based on materials hardness and tensile strength. Polyurethane plastic (Smooth Cast 310, Smooth-On Inc., Macungie, PA, USA) Shore D hardness of 70 and tensile strength of 3000 psi [26]. PC Rockwell R hardness of 118 and tensile strength >8900 psi [15].
